# Role of areca nut induced JNK/ATF2/Jun axis in the activation of TGF-β pathway in precancerous Oral Submucous Fibrosis

**DOI:** 10.1038/srep34314

**Published:** 2016-10-06

**Authors:** Ila Pant, S. Girish Rao, Paturu Kondaiah

**Affiliations:** 1 Department of Molecular Reproduction, Development and Genetics, Indian Institute of Science, Bangalore- 560012, India; 2 Department of Oral and Maxillofacial Surgery D.A Pandu Memorial- R.V Dental College and Hospital, Bangalore-560078, India

## Abstract

Oral submucous fibrosis (OSF) is potentially premalignant with progressive and irreversible extracellular matrix deposition accompanied by epithelial atrophy and like other fibrotic disorders, is primarily a TGF-β driven disease. OSF is caused by prolonged chewing of areca nut. Our previous studies reported a pivotal role for TGF-β activation and its effects contributing to OSF. However, the mechanism for activation of TGF-β signaling in OSF is still unknown. In this study we demonstrate activation of TGF-β signaling with sub-cytotoxic dose of areca nut in epithelial cells and discovered a key role for pJNK in this process. In good correlation; pJNK was detected in OSF tissues but not in normal tissues. Moreover, activation of JNK was found to be dependent on muscarinic acid receptor induced Ca^2+^/CAMKII as well as ROS. JNK dependent phosphorylation of ATF2/c-Jun transcription factors resulted in TGF-β transcription and its signaling. pATF2/p-c-Jun were enriched on TGF-β promoter and co-localized in nuclei of epithelial cells upon areca nut treatment. In corroboration, OSF tissue sections also had nuclear pATF2 and p-c-Jun. Our results provide comprehensive mechanistic details of TGF-β signaling induced by etiological agent areca nut in the manifestation of fibrosis which can lead to new therapeutic modalities for OSF.

Areca nut fruit of *Areca catechu* is widely chewed in the south-east Asian countries, East Africa and central Pacific to New Guinea with demographics estimating nearly 700 million people as consumers^1^,^2^. It is chewed in ripe or unripe; fresh or dried form. Areca nut is also consumed as a component of quid made with the leaf of *Piper betel* and slaked lime^1^,^3^. Areca nut has a complex mixture of alkaloids and polyphenols and its actions on physiological processes range from tachychardia, tremors to psychological effects like euphoria^3^,^4^,^5^,^6^. Reports suggest the addictive nature of areca nut wherein chewers suffer from withdrawal symptoms with discontinued usage^7^,^8^. Arecoline; an alkaloid component of areca nut has been classified as a class I carcinogen^3^. Amongst the wide ranging effects of areca nut on the human physiology; oral submucous fibrosis (OSF) disorder is prevalent in parts of globe where areca nut is consumed. Epidemiological studies and case reports suggest a direct correlation between prolonged areca nut chewing habit and development of potentially premalignant oral lesion; OSF^9^,^10^,^11^,^12^. OSF is a debilitating, inflammatory condition of the oral submucosa. It is characterized by oral epidermal thinning with concomitant increase in the extracellular matrix components leading to morbidity and trismus of the oral cavity^13^,^14^,^15^,^16^. In addition to the above mentioned characteristic features of OSF; histopathological evaluation of OSF cases reports epithelial dysplasia also^17^,^18^. Study conducted on 1 million retrospective Oral premalignant lesion cases reports a 10% conversion rate of OSF to oral squamous cell carcinoma (OSCC)^19^.

However, the mechanism for induction of fibrosis by areca nut is still unclear. Studies from our group and others implicated areca nut and its components in the regulation of genes like Transforming Growth Factor-β (TGF-β)^20^. Interleukin-6 (IL-6)^21^, Bone Morphogenetic Protein 7 (BMP7)^22^, collagen isoforms^23^ in the context of OSF. Activation of TGF-β pathway is central to development of organ fibrosis and its role in tumor promotion is well established^24^,^25^. Our earlier studies suggest TGF-β pathway activation in OSF tissues and by areca nut in epithelial cells. In these studies, we demonstrated induction of TGF-β2 by areca nut in keratinocytes^22^,^26^. In addition we also proposed that the TGF-β secreted from epithelial cells is essential for activation of fibroblasts; an outcome central to development of fibrosis. Moreover, gene expression profile induced by areca nut via TGF-β in epithelial cells and areca nut with TGF-β in fibroblasts was found to overlap with differential gene expression profile of OSF tissues^27^. Therefore, considering the importance and pivotal role of areca nut and TGF-β in the establishment of OSF; it is imperative to delineate how TGF-β is induced by areca nut in the disease process. It is unclear how areca nut; a plant derived extract can activate signaling pathways in epithelial cells which results in up-regulation of the pro-fibrotic TGF-β/SMAD pathway in OSF. Therefore, here we studied the regulation of TGF-β signaling by areca nut in epithelial cells and demonstrate the mechanism of TGF-β2 induction and pathway activation by areca nut in epithelial cells involving activation of JNK/ATF2/Jun axis and show their relevance in OSF.

## Results

### Activation of TGF-β pathway by areca nut

**Figure 1:**
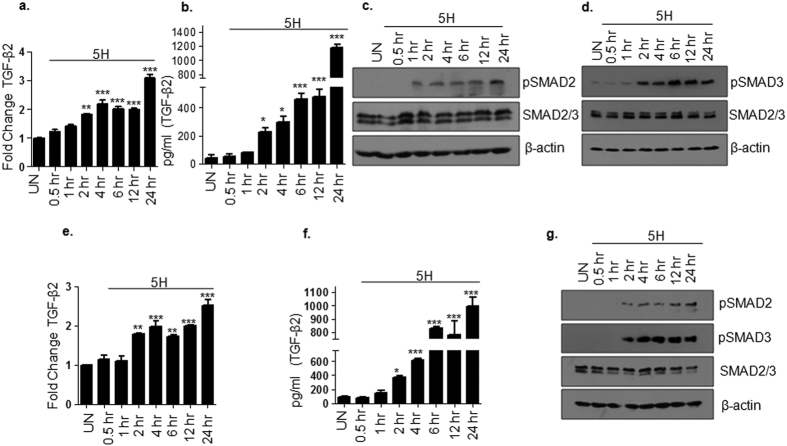
Activation of TGF-β pathway by areca nut in epithelial cells. (**a**) qRT-PCR data representing fold change in TGF-β2 transcript in HaCaT cells upon areca nut treatment (5H; 5 μg/ml) at various time points compared to untreated cells. (**b**) Bar graph representing TGF-β2 protein in HaCaT cells induced by areca nut treatment (5H; 5 μg/ml) at various time points compared to untreated cells. (**c,d**) Immunoblots depicting areca nut (5H; 5 μg/ml) induced phosphorylation of SMAD2 (**c**) and SMAD3 (**d**) in HaCaT cells at various time points compared to untreated cells. (**e**) qRT-PCR data representing fold change in TGF-β2 transcript in HPL1D cells upon areca nut treatment (5H; 5 μg/ml) at various time points compared to untreated cells. (**f**) Bar graph representing TGF-β2 protein in HPL1D cells induced by areca nut treatment (5H; 5 μg/ml) at various time points compared to untreated cells. (**g**) Immunoblots representing areca nut (5H; 5 μg/ml) induced phosphorylation of SMAD2 and SMAD3 in HPL1D cells at various time points compared to untreated cells. β-actin is used as loading control in all the immunoblots. ***^,^**^,^* represent p values ≤ 0.0001, 0.001, 0.01 respectively.

Areca nut treatment (5H; 5 μg/ml) was given to epithelial cells (HaCaT and HPL1D) for various time points (0.5, 1, 2, 4, 6, 12 and 24 hours) to determine the kinetics of TGF-β pathway activation. TGF-β2 mRNA was induced by areca nut post 2 hour treatment with a concomitant sustained increase in TGF-β2 protein and activation of canonical downstream effectors, SMAD2 and 3 (pSMAD2 and 3) in HaCaT (Fig. 1a–d) as well as HPL1D cells (Fig. 1e–g). These SMAD proteins are also known to be activated by other TGF-β superfamily of ligands namely; TGF-β1, TGF-β3, Activin A, Activin B and Nodal. Therefore, we also tested whether areca nut regulates the transcripts of these genes. Areca nut induced TGF-β1, INHBA (Activin A), INHBB (Activin B) at late time points (post 4 hours of areca nut treatment) in HaCaT (Supplementary Fig. 1a–d) and HPL1D cells (Supplementary Fig. 1e,g,h). While TGF-β3 transcript did not show significant up-regulation in HaCaT cells (Supplementary Fig. 1b); it was up-regulated in HPL1D cells at 24 hour areca nut treatment (Supplementary Fig. 1f). Transcript for Nodal was not detectable in both HaCaT and HPL1D cells (data not shown). These data demonstrated that areca nut induced canonical SMAD pathway activation coincided with the up-regulation of TGF-β2 transcript at 2 hours. Hence, we further pursued the mechanism of areca nut mediated regulation of TGF-β2 transcript and SMAD pathway induction in epithelial cells.

**Figure 2:**
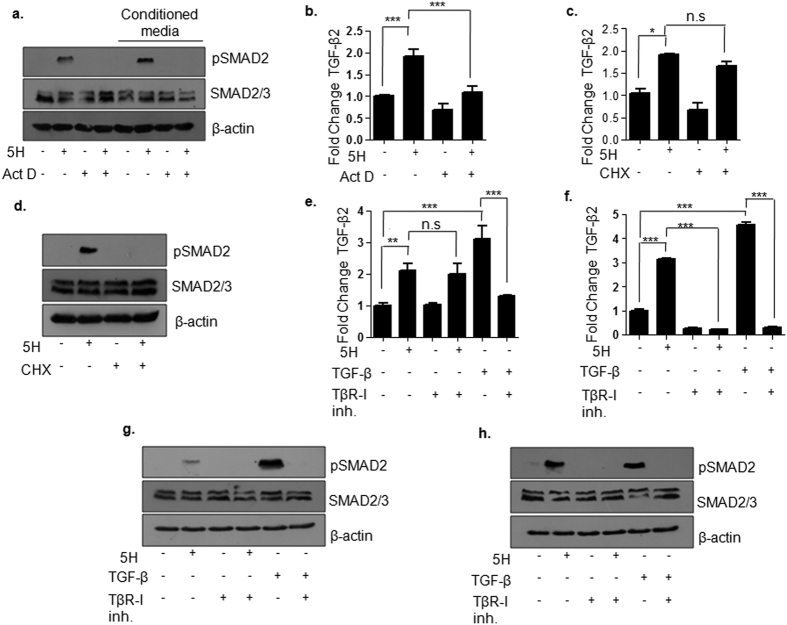
Areca nut mediated activation of TGF-β pathway is dependent on transcription in HaCaT cells. (**a**) Immunoblot of HaCaT extracts probed with pSMAD2 antibodies. First four lanes depict pSMAD2 levels upon 2 hour treatment with areca nut with or without actinomycin D. Last four lanes of the immunoblot depict pSMAD2 levels upon further treatment of untreated serum starved HaCaT cells for 2 hours with conditioned media of areca nut treated cells with or without actinomycin D (Act D). (**b**) TGF-β2 transcript levels at 2 hours post areca nut and/or actinomycin D (Act D) treatment. (**c,d**) TGF-β2 transcript and pSMAD2 levels at 2 hours post areca nut and/or cycloheximide (CHX) treatment. TGF-β2 transcript at 2 hours (**e**) and 24 hours (**f**) post areca nut or TGF-β with or without TβR-I inhibitor treatment. pSMAD2 levels at 2 hours (**g**) and 24 hours (**h**) post areca nut or TGF-β with or without TβR-I inhibitor treatment. β-actin is used as loading control in all the immunoblots. (5H; 5 μg/ml areca nut extract). ***^,^**^,^* represent p values ≤ 0.0001; 0.001; 0.01 respectively.

This regulation of TGF-β2 by areca nut could be due to direct activation of transcription from the TGF-β2 promoter or activation of the latent TGF-β2 protein resulting in the auto-induction. In both these cases the ligand can bind to the cognate receptor to initiate SMAD signaling. Therefore, both these possibilities were tested in HaCaT cells by the following experiments. HaCaT cells were pre-treated with actinomycin D in order to block transcription; followed by areca nut treatment for 2 hours. Furthermore, the conditioned media of the cells with the treatments mentioned above was also used to treat serum deprived HaCaT cells for 2 hours. As depicted in Fig. 2a; areca nut induced pSMAD2 was compromised in the presence of actinomycin D. Additionally, conditioned media of areca nut treated cells also induced pSMAD2 at 2 hours but this induction was not observed with the treatment of conditioned media from areca nut and actinomycin D treated cells. Therefore, activation of SMAD2 by areca nut requires active transcription. Hence, we tested transcriptional regulation of TGF-β2 by areca nut treatment in the presence of actinomycin D at 2 hours. Areca nut failed to induce TGF-β2 mRNA in the presence of actinomycin D (Fig. 2b), suggesting requirement of active transcription for this induction. Interestingly, areca nut treatment for 2 hours was able to induce TGF-β2 transcript in the presence of translational inhibitor, cycloheximide (Fig. 2c) but not activation of SMAD2 (pSMAD2) at this time point (Fig. 2d). We further corroborated these results in HPL1D cells. As shown in Supplementary Fig. 2a, areca nut induced TGF-β2 transcript at 2 hours was also found to be compromised in the presence of actinomycin D but not in presence of cycloheximide in HPL1D cells. Also, induction of pSMAD2 by areca nut at 2 hours was abrogated in the presence of actinomycin D as well as cycloheximide (Supplementary Fig. 2b). These data therefore indicated that areca nut induced TGF-β2 transcription is independent of the protein translation.

It is well established that TGF-β transcripts can be self-regulatory and auto-induced^28^. Hence, we tested the requirement for TGF-β receptor I (TβR-I) for induction of TGF-β2 transcript by areca nut. For this we used an inhibitor of TβR-I, SB431542^29^. As shown in Fig. 2e; areca nut induced TGF-β2 mRNA at 2 hours was found to be independent of TβR-I. A similar experiment was performed at 24 hours wherein treatment of areca nut was given in the presence or absence of SB431542. At this time point; induction of TGF-β2 transcript by areca nut was found to be dependent on TβR-I (Fig. 2f). However, activation of SMAD2 by areca nut was found to be dependent on TβR-I at both 2 and 24 hour time points (Fig. 2g,h). This led us to conclude that the first wave of TGF-β2 mRNA induction by areca nut is independent of TβR-I and subsequent regulation requires TβR-I activation.

Therefore, all these data indicated that activation of SMAD pathway by areca nut requires fresh transcription, translation as well as TβR-I activity. However, the initial induction of TGF-β2 mRNA by areca nut is independent of active protein translation, activation of pre-existing latent TGF-β and TβR-I activity.

### TGF-β2 induction and pathway activation by areca nut is via JNK

**Figure 3:**
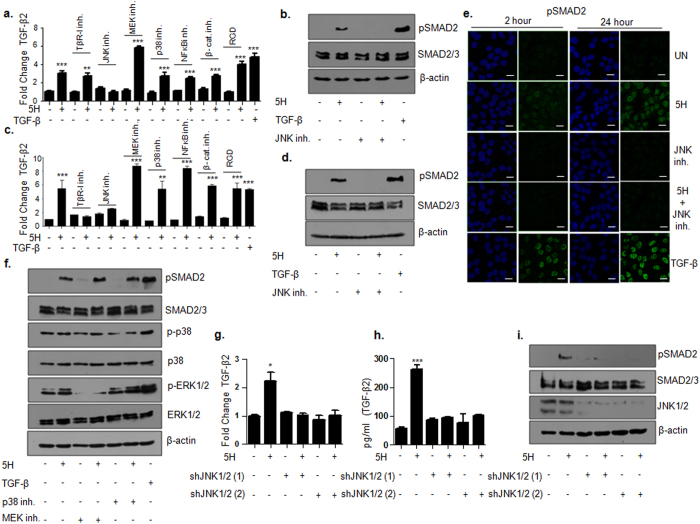
Activation of TGF-β pathway by areca nut is dependent on JNK in HaCaT cells. (**a,b**) Regulation of TGF-β2 transcript (bar graph) and pSMAD2 (immunoblot) respectively by 5H treatment with or without the indicated inhibitors at 2 hour time point. (**c,d**) Regulation of TGF-β2 transcript (bar graph) and pSMAD2 (immunoblot) respectively by 5H treatment with or without the indicated inhibitors at 24 hour time point. (**e**) Immunofluorescence data depicting induction of nuclear localized pSMAD2 by 5H at 2 and 24 hours which is compromised in the presence of JNK inhibitor. (**f**) Immunoblot representing pSMAD2 levels upon 24 hour treatment of 5H with or without MEK or p38 inhibitor. Levels of p-p38, pERK1/2 served as positive controls for the inhibitors. TGF-β treatment is used as positive control in (**a–f**). Graphs respresenting TGF-β2 transcript (**g**) and protein (**h**) at 2 hours upon transient knockdown of JNK1/2 by using two different combinations of shRNAs (1 & 2) with or without areca nut treatment. (**i**) Immunoblot representing pSMAD2 at 2 hours upon transient knockdown of JNK1/2 using two different combinations of shRNAs (1 & 2) with or without areca nut treatment. β-actin is used as loading control in all the immunoblots. ***^,^**^,^* represent p values ≤ 0.0001; 0.001; 0.01 respectively. (5H; 5 μg/ml areca nut extract).

Since, the initial induction of TGF-β2 by areca nut was found to be independent of TβR-I; we explored the role of other pathways in areca nut mediated TGF-β2 induction in HaCaT cells. Areca nut failed to induce TGF-β2 mRNA upon inhibition of JNK activity by SP600125 (small molecule inhibitor) whereas inhibitors of MEK/ERK, p38, NFκB, β-catenin/TCF, Integrin (RGD) could not compromise areca nut induced TGF-β2 transcript at 2 (Fig. 3a) and 24 hour (Fig. 3c) time points. In line with this, areca nut could not induce pSMAD2 in the presence of JNK inhibitor at both 2 hours (Fig. 3b) and 24 hours (Fig. 3d). This observation was confirmed by immunocytochemistry for pSMAD2 induction in cells treated with areca nut (with or without JNK inhibitor) at 2 and 24 hour time points. Areca nut induced nuclear localization of pSMAD2 but was not able to induce pSMAD2 in the absence of JNK activity at both the time points (Fig. 3e). In addition, abrogation of ERK1/2 and p38 activity did not have any effect on areca nut induced pSMAD2 at 24 hours (Fig. 3f). We further corroborated these results by transient knockdown of JNK1 and 2 in HaCaT cells using two different shRNAs each and studied areca nut induced TGF-β2 mRNA, protein and pSMAD2 at 2 hours. JNK1/2 knockdown significantly compromised the induction of TGF-β2 transcript, protein and pSMAD2 by areca nut (Fig. 3g–i).

Similar experiments were also performed in HPL1D cells. In these cells also, areca nut induced TGF-β2 mRNA was compromised by JNK inhibition at both 2 and 24 hours. Inhibition of other pathways mentioned above did not abrogate this induction (Supplementary Fig. 3a,b). Further, knockdown of JNK1/2 by shRNAs compromised areca nut induced TGF-β2 mRNA, protein and pSMAD2 (Supplementary Fig. 3c–e). These results suggest that JNK initiates the first induction of TGF-β2 transcription by areca nut which is subsequently followed by auto-regulation of TGF-β2 transcript and pathway activation.

**Figure 4:**
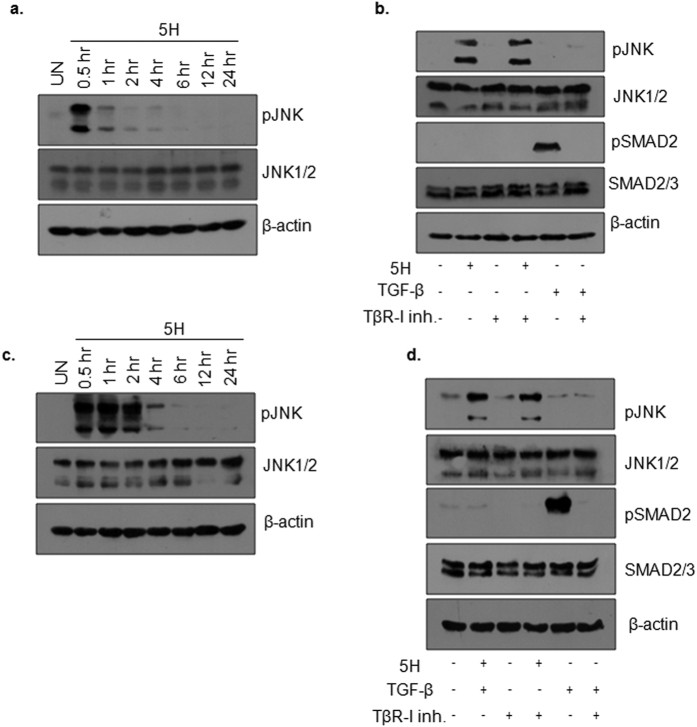
Areca nut activates JNK in epithelial cells. (**a**) Immnunoblot representing kinetics of pJNK induction by 5H in HaCaT cells at the indicated time points. (**b**) Immunoblot representing pJNK and pSMAD2 levels upon 0.5 hour treatment of 5H or TGF-β with or without TβR-I inhibitor on HaCaT cells. (**c**) Immnunoblot representing kinetics pJNK induction by areca nut in HPL1D cells at the indicated time points. (**b**) Immunoblot representing pJNK and pSMAD2 levels upon 0.5 hour treatment of 5H or TGF-β with or without TβR-I inhibitor on HPL1D cells. β-actin is used as loading control in all the immunoblots. (5H; 5 μg/ml areca nut extract, TβR-I inhibitor; 10 μM TGF-β receptor I inhibitor).

We also established the timeline for areca nut induced pJNK in epithelial cells. Areca nut treatment induced pJNK maximally at 0.5 hour in HaCaT cells and was undetectable after 4 hours (Fig. 4a). Importantly, induction of pJNK by areca nut in these cells was independent of TβR-I activity (Fig. 4b). We also validated these results in HPL1D cells. Areca nut induced pJNK in these cells at 0.5 hours, which sustained till 2 hours and was not detected post 4 hour treatment (Fig. 4c). This up-regulation of pJNK was also found to be independent of TβR-I activity (Fig. 4d).

Taken together, these data establish the activation of JNK by areca nut as well as its requirement for areca nut mediated TGF-β signal activation in epithelial cells.

### Areca nut induced activation of JNK/TGF-β axis is dependent on Ca^2+^/CAMKII and ROS

**Figure 5:**
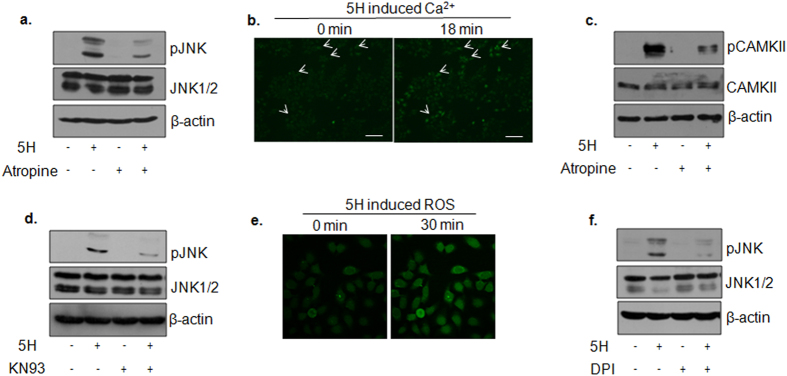
Areca nut induced JNK activation is dependent on Ca^2+^/CAMKII and ROS in HaCaT cells. (**a**) Immunoblot representing compromise in areca nut induced pJNK levels in the presence of muscarinic acid receptor inhibitor (atropine) at 0.5 hours. (**b**) Representative images from Supplementary video 1 depicting areca nut induced calcium release. White arrows depict the cells with higher calcium levels post areca nut treatment. Magnification factor; 10X, Scale bar = 100 μm. (**c**) Immunoblot representing compromise in areca nut induced pCAMKII levels in the presence of muscarinic acid receptor inhibitor (atropine) at 0.5 hours. (**d**) Immunoblot representing compromise in areca nut induced pJNK levels in the presence of CAMKII inhibitor (KN93) at 0.5 hours. (**e**) Representative images for live confocal imaging of areca nut induced ROS at 0 and 30 minutes. Magnification factor; 63X. (**f**) Immunoblot representing compromise in areca nut induced pJNK levels in the presence of ROS inhibitor (DPI) at 0.5 hours. β-actin is used as loading control in all the immunoblots.

Given the importance of JNK activation in areca nut mediated TGF-β signal activation, we further explored the areca nut regulated upstream modulators of JNK in epithelial cells. Arecoline is a major alkaloid component of areca nut and is a known muscarinic acid receptor agonist^30^. Therefore, we tested the possibility of the muscarinic acid receptor involvement in areca nut induced pJNK. Inhibition of this receptor by atropine led to a compromise in areca nut induced pJNK in HaCaT cells (Fig. 5a). Activation of muscarinic acid receptor is also known to increase intracellular Ca^2+^ and CAMKII activity^31^. Hence the possibility for activation of Ca^2+^and CAMKII by areca nut was explored. As shown in Fig. 5b, areca nut increased intracellular calcium mobilization within 18 minutes of treatment on HaCaT cells (also see Supplementary video 1). In line with this, 0.5 hour treatment of areca nut increased pCAMKII which was compromised upon inhibition of muscarinic acid receptor by atropine in HaCaT cells (Fig. 5c). Further, areca nut failed to activate JNK at 0.5 hours upon abrogation of CAMKII activity by KN93 (small molecule inhibitor) (Fig. 5d).

Reactive oxygen species (ROS) is also known to activate JNK^32^. Hence we tested the role of ROS in the activation of JNK by areca nut. Areca nut was able to induce ROS within 30 minutes of treatment on HaCaT cells (Fig. 5e). Also, inhibition of ROS generation by DPI significantly compromised areca nut induced pJNK at 0.5 hours in these cells (Fig. 5f).

JNK activation is also reported to be regulated by Rac-GTPase and TAK1^33^,^34^,^35^. Interestingly, inhibition of Rac-GTPase did not decrease areca nut induced pJNK in HaCaT cells (Supplementary Fig. 4a). Also, 0.5 hour treatment of HaCaT cells with areca nut did not induce pTAK1 (Supplementary Fig. 4b). Moreover, pJNK induction by areca nut in HaCaT cells was not compromised by over-expression of dominant negative TAK1 or transient knockdown of TAK1 using two different shRNAs (Supplementary Fig. 4c,d).

**Figure 6:**
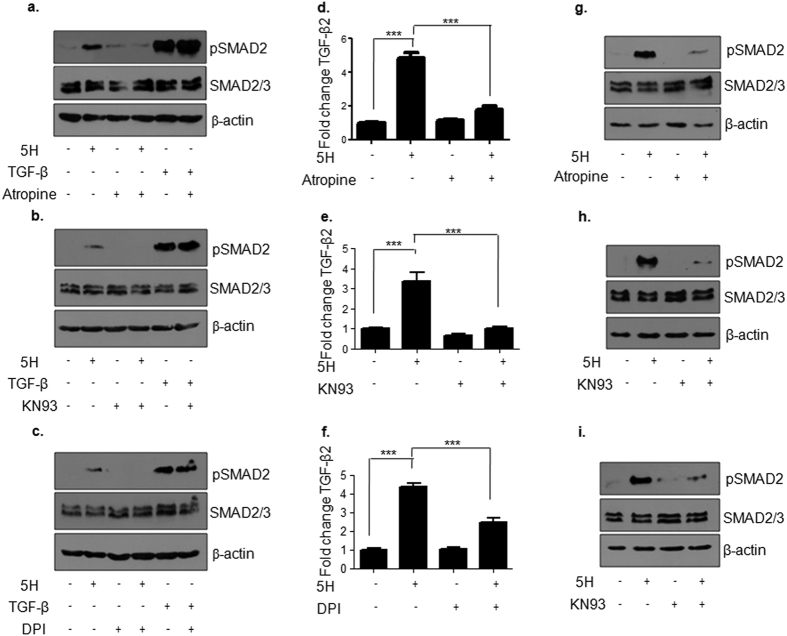
Activation of TGF-β signaling by areca nut is dependent on Ca^2+^/CAMKII and ROS in HaCaT cells. (**a–c**) Immunoblots representing pSMAD2 levels upon 2 hour treatment of areca nut or TGF-β with or without the indicated inhibitors (atropine, KN93 and DPI). (**d–f**) Bar graphs showing induction of TGF-β2 transcript upon 24 hour treatment of areca nut which is compromised in the presence of the indicated inhibitors (atropine, KN93 and DPI). (**g–i**) Immunoblots representing pSMAD2 levels upon 24 hour treatment of areca nut with or without the indicated inhibitors (atropine, KN93 and DPI). β-actin is used as loading control in all the immunoblots. Error bars represent mean ± SEM from n = 3. p ≤ 0.01, 0.001, 0.0001 is represented as *^,^**^,^*** respectively. (5H; 5 μg/ml areca nut extract).

As muscarinic acid receptor, CAMKII and ROS were found to regulate areca nut induced pJNK levels; we further tested whether inhibition of any of these three can regulate areca nut induced TGF-β signaling. Upon compromising muscarinic acid receptor and CAMKII activity or ROS generation by atropine, KN93 and DPI respectively; areca nut failed to induce pSMAD2 at 2 hours whereas TGF-β induced pSMAD2 was unaffected in the presence of these inhibitors (Fig. 6a–c). We further tested these results at 24 hour time point by the inhibition of these three molecules. At this time point also; areca nut failed to induce TGF-β2 transcript (Fig. 6d–f) and pSMAD2 (Fig. 6g–i).

These results were further validated using HPL1D cells. Treatment of HPL1D cells by atropine, KN93 and DPI compromised areca nut mediated up regulation of pJNK suggesting the involvement of muscarinic acid receptor, CAMKII and ROS in this regulation (Supplementary Fig. 5a,c,d). Regulation of pCAMKII by areca nut through muscarinic receptor is shown in supplementary Fig. 5b. Moreover, induction of pJNK by areca nut was found to be independent of Rac-GTase and TAK1 at 0.5 hour (Supplementary Fig. 5e,f). Finally, inhibition of muscarinic acid receptor, CAMKII and ROS compromised areca nut induced TGF-β2 mRNA and pSMAD2 in these cells at 2 hours (Supplementary Fig. 5g,h).

### Areca nut activates ATF2 and c-Jun

**Figure 7:**
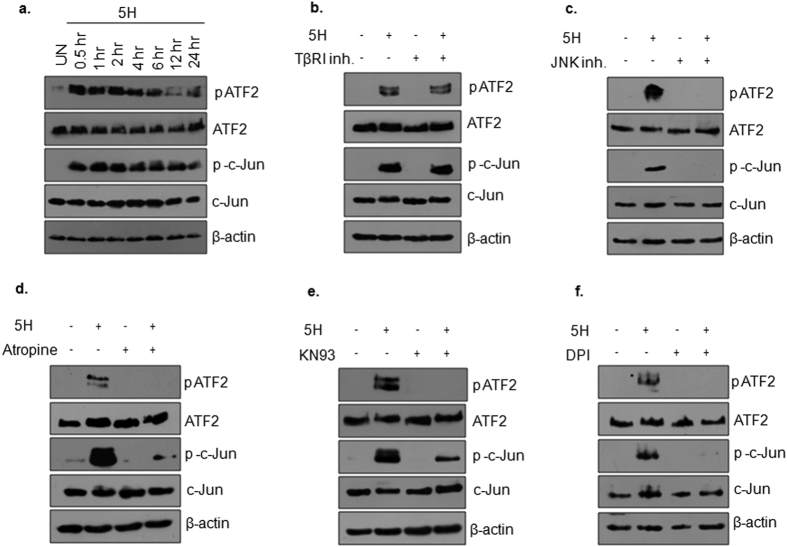
Areca nut activates ATF2 and Jun in HaCaT cells. (**a**) Protein expression data for phospho and total proteins of ATF2 and c-Jun upon areca nut treatment for the indicated time points. (**b,c**) Immunoblots depicting induction of phospho ATF2 and phospho c-Jun by areca nut is independent of TβR-I (**b**) but dependent on JNK (**c**) activity. (**d–f**) Immunoblots representing compromise in areca nut induced pATF2/p-c-Jun upon treatment with (**d**) atropine; (**e**) KN93 and (**f**) DPI. β-actin is used as loading control in all the immunoblots.

Mechanism of JNK induced TGF-β pathway activation was further investigated. Reports in literature suggest ATF2 and AP1 complexes mediate autocrine regulation of TGF-β production in various cell types^28^,^36^,^37^,^38^. Therefore, we tested activation of these transcription factors upon areca nut treatment for various time points (0.5, 1, 2, 4, 6, 12 and 24 hours) in HaCaT cells. pATF2 and p-c-Jun were consistently up-regulated by areca nut treatment (Fig. 7a). The activation of ATF2 and c-Jun was found to be dependent on JNK but independent of TβR-I activity (Fig. 7b,c). Therefore, we tested if regulators of JNK have any effect on areca nut induced pATF2 and p-c-Jun at 0.5 hours. The activation of ATF2 and c-Jun by areca nut was found to be compromised by muscarinic acid receptor, CAMKII and ROS inhibitors (Fig. 7d–f).

We further corroborated our observations using HPL1D cells. Sustained phosphorylation of ATF2 and c-Jun was observed following areca nut treatment for all time points tested (Supplementary Fig. 6a) and this regulation was also found to be independent of TβR-I but dependent on JNK activity (Supplementary Fig. 6b).

### ATF2/c-Jun mediate areca nut induced TGF-β pathway activation

**Figure 8:**
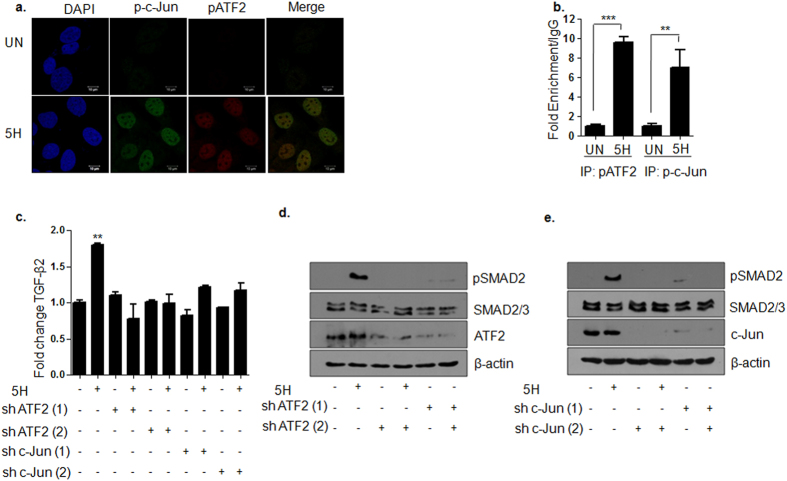
ATF2/Jun mediate areca nut induced TGF-β pathway activation in HaCaT cells. (**a**) Representative immunofluorescence images of areca nut induced nuclear localized pATF2 and p-c-Jun at 2 hours. Magnification: 63X; Zoom: 1.8; Scale bar = 10 μm. (**b**) Bar graph of qPCR results of chIP assay showing fold enrichment of pATF2 and p-c-Jun on TGF-β2 promoter at 2 hours upon areca nut treatment compared to untreated cells. (**c**) Bar graph showing fold change at 2 hour of areca nut induced TGF-β2 and its compromise upon transient knock down of ATF2 or c-Jun. (**d,e**) Immunoblots representing pSMAD2 levels at 2 hour areca nut treatment and its compromise upon transient knock down of ATF2 or c-Jun. β-actin is used as loading control in all the immunoblots. ***^,^** represent p values ≤ 0.0001; 0.001 respectively. (5H; 5 μg/ml areca nut extract).

As discussed earlier, both ATF2 and c-Jun were activated upon areca nut treatment which was found to be dependent on JNK. We further evaluated the localization and significance of these phospho proteins in the context of TGF-β pathway activation by areca nut. 2 hour areca nut treatment on HaCaT cells resulted in co-localization of pATF2 and p-c-Jun in the nucleus (Fig. 8a). Therefore, we then tested the TGF-β2 promoter occupancy of these two transcription factors by chIP assay upon 2 hour areca nut treatment. Immunoprecipitation by pATF2 or p-c-Jun antibodies followed by PCR amplification with specific primer pair indicated enriched amplification of the region between −36 to −148 of the TGF-β2 promoter, upon areca nut treatment (Fig. 8b). This region harbors CRE/ATF consensus binding sequence from −63 to −71^39^ in close proximity to AP1 like elements^40^ suggesting binding of ATF2 and c-Jun to these elements.

To further assess which of the two transcription factors may be essential for areca nut induced TGF-β signal activation, ATF2 or c-Jun were transiently knockdown by using two different shRNAs. The regulation of TGF-β2 transcript and pSMAD2 by areca nut at 2 hours was further assessed in this background. Knockdown of ATF2 and c-Jun compromised areca nut regulated induction of TGF-β2 transcript (Fig. 8c) and pSMAD2 (Fig. 8d,e) in HaCaT cells thereby confirming the role of both these transcription factors in the induction of TGF-β signaling in response to areca nut treatment.

These results were also validated in HPL1D cells. Areca nut treatment for 2 hours on HPL1D cells enriched pATF2 and p-c-Jun on TGF-β2 promoter similar to HaCaT cells (Supplementary Fig. 7a). Further, transient knockdown of ATF2 and c-Jun in these cells also compromised areca nut induced TGF-β2 transcript (Supplementary Fig. 7b) and pSMAD2 at 2 hour time point (Supplementary Fig. 7c,d). All these data suggest that ATF2 and c-Jun transcription factors are essential in areca nut mediated TGF-β signaling in epithelial cells.

### JNK/ATF2/Jun axis is activated in OSF tissues

**Figure 9:**
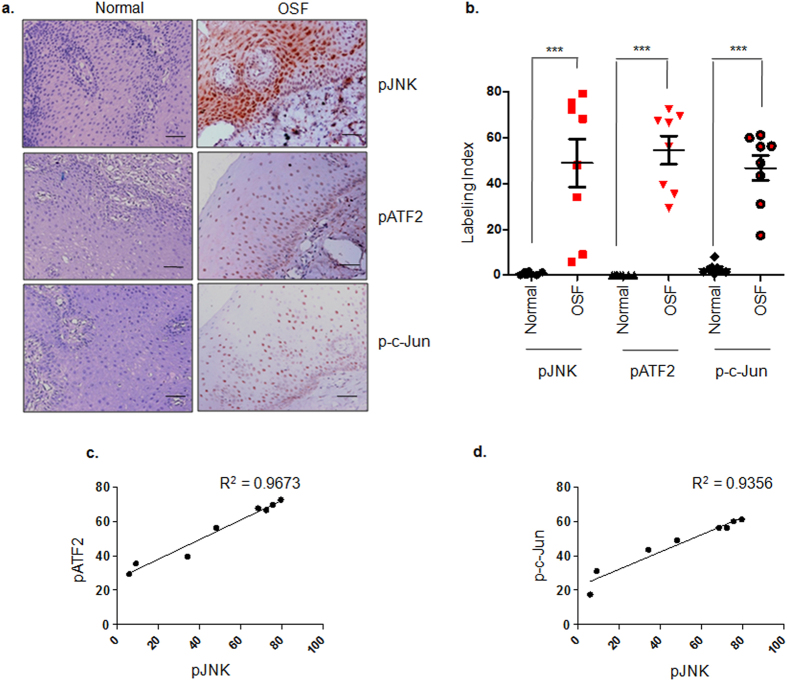
pJNK, pATF2 and p-c-Jun are up regulated in OSF tissues. (**a**) Representative images of immunohistochemistry performed on normal (n = 8) and OSF (n = 8) tissues for evaluating activation of JNK (pJNK), ATF2 (pATF2) and c-Jun (p-c-Jun). pJNK, pATF2 and p-c-Jun levels were observed to be high in OSF tissues as compared to normal tissues. Magnification factor = 20X; scale bar = 50 μm. (**b**) Scatter graph depicting labeling index for each of the tissue samples scored for pJNK, pATF2 and p-c-Jun staining. Unpaired t-test was performed for statistical significance between the median labeling index of two groups (Normal and OSF tissues). (**c**) Pearson’s correlation graph for pATF2 (Y axis) and pJNK (X axis) staining in OSF tissues. (**d**) Pearson’s correlation graph for p-c-Jun (Y axis) and pJNK (X axis) staining in OSF tissues. *** represents p value ≤ 0.0001.

Activation of TGF-β signaling has been previously reported by our group wherein pSMAD2 was observed to be localized in the nucleus of epithelial cells of OSF tissues and was not detected in normal tissues^22^. In the present study; *in vitro* data indicated JNK dependent activation of ATF2 and c-Jun to be essential for areca nut mediated TGF-β signaling in epithelial cells. Therefore, we assessed the status of phospho forms of JNK, ATF2 and c-Jun in OSF tissues and compared it to the staining in normal tissues. pJNK levels were found to be up-regulated in OSF tissues (n = 8) with the median labeling index (LI) of 48.02% while its activation was not observed in normal tissues (n = 8; median LI = 0.0483%) (Fig. 9a,b). In good correlation, both pATF-2 and p-c-Jun were found to be localized in the nucleus of epithelial cells of OSF tissues (Median LI = 61.47% and 52.59%; respectively) whereas their expression was not detected in normal tissues (Median LI of pATF2 = 0.007%; Median LI p-c-Jun = 2.135%) (Fig. 9a,b). We further performed Pearson’s correlation analysis for pJNK/pATF2 and pJNK/p-c-Jun based on the labeling index. Both pATF2 and p-c-Jun were found to be positively correlated with pJNK with Pearson’s correlation index (Pearson’s r) as 0.9835 and 0.9672, respectively and p values < 0.0001(Fig. 9c,d). Therefore, these data establish the activation of JNK/ATF2/Jun axis in OSF tissues.

## Discussion

The current study stems from lacunae in the understanding of molecular mechanisms of OSF manifestation. As described in the introduction, TGF-β pathway activation in epithelial cells is an important event in OSF^22^,^26^,^27^. However, there have been no reports addressing the mechanism behind activation of TGF-β pathway. Towards this, we have utilized two epithelial cell lines namely, HaCaT and HPL1D. In this study we demonstrate that areca nut induces TGF-β2 transcript, protein as well as the SMAD pathway within two hours of treatment on epithelial cells and the induction of the transcript at this time point is independent of TβR-I. This data was unexpected; as results from our previous studies performed with 48 hour areca nut treatment had consistently shown dependency of TGF-β2 transcript induction/SMAD activation on TβR-I^26^,^27^. We further tested the regulation of SMAD activation by areca nut at 2 and 24 hour time points and found it to be dependent on TβR-I activity at both the time points. This result gave a clue that areca nut up regulates TGF-β2 transcript at early time point by activation of some other pathway other than canonical TGF-β pathway. Based on this, we hypothesized that after initial induction of TGF-β2, it then can auto-regulate its induction^28^; which is probably why we observed a compromise of TGF-β2 transcript with the use of TβR-I inhibitor at later time points.

By using a panel of inhibitors we further found JNK activation to be essential for TGF-β pathway activation. Areca nut extract is known to activate MAPK’s (ERK1/2; JNK and p38) and NFκB^41^. But the implication for the activation of these molecules in OSF has not been addressed. None of the other MAPKs (ERK1/2; p38) or other pathways were found to be involved in TGF-β2 induction by areca nut in our study; thereby emphasizing the importance of JNK activation. This is in agreement with the report on the requirement of JNK activation during carbon tetrachloride induced liver fibrosis^42^. JNK activation in response to TGF-β in dermal fibrosis is also reported^43^. Moreover, there are ongoing clinical trials for CC-930; an orally administered JNK inhibitor as a therapeutic option for idiopathic pulmonary fibrosis^44^. However, our study proposes JNK activation in OSF as an essential event up stream to TGF-β. In light of this, JNK activation by areca nut may be an important event in the initiation of OSF.

Upon further investigation, we were able to identify a role for areca nut induced muscarinic acid receptor mediated Ca^2+^/CAMKII in JNK induction. One of the reports attributes tracheal hyper-responsiveness in bleomycin induced pulmonary fibrosis and circulating IL-1β levels to muscarinic acid receptor activation and intracellular calcium increase^45^. Our study also suggests involvement of muscarinic acid receptors in the activation of JNK in OSF. However, further investigations are required to establish the role of muscarinic acid receptor activation in the context of OSF and other fibrotic disorders. There are several studies that provide compelling evidence for the role of ROS in various fibrotic disorders^46^,^47^,^48^,^49^. ROS mediated activation of MAPKs is an established phenomenon but the mechanism is still elusive^32^. In our study, we observed ROS induction within 30 minutes of areca nut treatment and attenuation of ROS generation compromised areca nut induced pJNK. Since activation of muscarinic acid receptor/Ca^2+^/CAMKII and ROS were regulated by areca nut thereby activating JNK; we further tested whether compromising each of them had an impact on TGF-β signaling. Attenuation of any of these molecules abrogated TGF-β2 and downstream pSMAD2 induction. Rac- GTPases and TAK1 are other molecules known to regulate JNK activity^33^,^34^,^35^. But in our study, inhibition of these two did not have any effect on the activation of JNK by areca nut suggesting JNK activation is independent of both Rac and TAK.

After establishing that JNK activation by areca nut is a pre-requisite to TGF-β signaling, we explored which probable JNK effector transcription factors could lead to TGF-β signaling by areca nut. Several reports suggest the role of transcription factors belonging to AP1 family in TGF-β regulation^37^,^38^. AP1 family members have a bZIP domain which allows them to homo/heterodimerize with each other. The transcription program regulated by these factors is dependent on the binding partner (s)^50^. Among the Jun family members (c-Jun, JunB, JunD), c-Jun phosphorylation at Ser 63/73 by JNK is well known; whereas JunB does not have JNK mediated phospho-accepting sites. JunD does not have JNK docking site which results in weak phosphorylation and ERK1/2 mediated phosphorylation is stronger^51^,^52^. ATF2 is another known JNK target and gets phosphorylated at Thr 69/71 leading to its active conformation thereby allowing it to partner with other AP1 members like c-Jun^52^. Moreover, in a chromatin immunoprecipitation screen; genotoxic stress was found to enrich c-Jun and ATF2 transcription factors on TGF-β promoter^53^. All these studies point to a plausible activation of c-Jun and ATF2 in response to areca nut; which is a known cellular stress^21^,^54^. In this study we have identified areca nut induced JNK mediated activation of ATF2 and c-Jun transcription factors. This chain of events leads to TGF-β2 transcription thereby canonical SMAD pathway activation. Up-regulation of pSMAD2 in OSF tissues has been previously published by our group^22^. In this study, we observed activation of both ATF2 and c-Jun in epithelial cells of OSF patients which was absent in normal subjects. c-Jun is a widely expressed protein in squamous cells but its activation; if observed is usually in the mitotically active basal epithelial cells of normal oral mucosa. ATF2 protein on the other hand is not highly expressed in oral mucosa (www.proteinatlas.org). But, the expression pattern of pATF2 and p-c-Jun in OSF tissues in this study displayed expression in basal epithelial cells; stratum spinosum; stratum superficiale as well as endothelial cells. This expression data correlates positively with the pJNK status in the OSF tissues (Fig. 9c,d).

Over-expression of TGF-β, activation of c-Jun and ATF2 is well established in several cancers^25^,^52^. Moreover, an immunohistochemical study found nuclear p-c-Jun in 60% of their cohort of OSCC tissues and negative staining in normal tissues^55^. Our observation is consistent with the dysplastic/hyperplastic changes observed in some OSF cases. Up-regulation of all the three above mentioned molecules in OSF (premalignant lesion); is a concern since a significant proportion of OSF patients develop OSCC^19^. Genetic predisposition to oral cancer with concomitant increase in TGF-β and oncogenic molecules such as c-Jun; ATF2 in OSF may signify progression to oral squamous cell carcinoma (OSCC). In view of this, intervention of this axis may provide better strategy to prevent development of OSCC in OSF patients. This needs further investigations.

**Figure 10:**
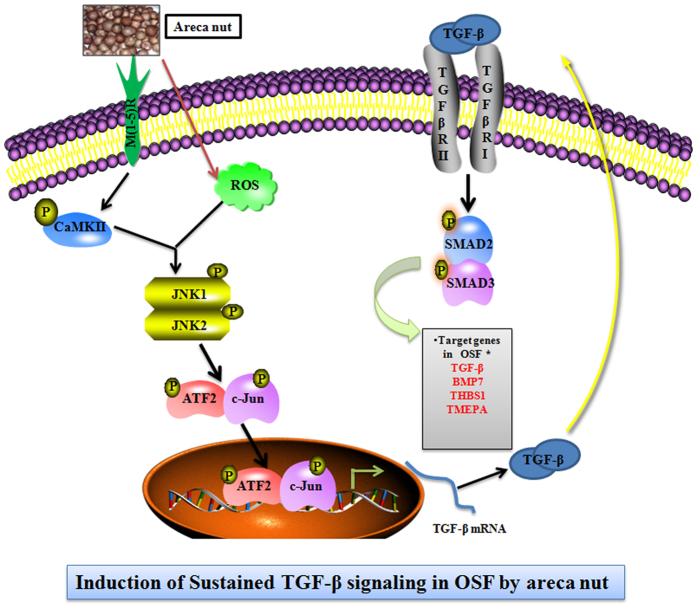
Schematic representation of key findings from the study. Areca nut acts on muscarinic acid receptors to release calcium and activate CAMKII. It also induces intracellular reactive oxygen species (ROS). CAMKII and ROS together activate JNK which subsequently phosphorylates ATF2 and c-Jun transcription factors. The two transcription factors induce TGF-β2 promoter. The translated TGF-β protein can now activate the canonical SMAD signaling pathway and auto-induce TGF-β and other targets *^22^ in epithelial cells.

In conclusion, we provide mechanistic details of TGF-β pathway activation in OSF. This is the first report in OSF invoking involvement of JNK/ATF2/Jun in TGF-β pathway activation by the etiological agent; areca nut. Our data, demonstrates the activation of JNK by areca nut via muscarinic acid receptor/Ca^2+^/CAMKII and ROS. Phospho-JNK then phosphorylates and activates ATF2; c-Jun. These two transcription factors activate transcription from the TGF-β promoter. The TGF-β protein thus translated can activate canonical pathway via TGF-β type I receptor. This leads to a sustained TGF-β autocrine loop which promotes OSF and up-regulation of other pro-fibrotic genes (Fig. 10). In view of very few options for the therapeutic management of OSF that includes anti-inflammatory agents and surgical resection, our study which highlights the importance of signaling pathways regulated by areca nut may provide better strategies for the management of OSF and subsequent prevention of conversion to OSCC.

## Materials and Methods

### Areca nut extract preparation

Areca nut extract was prepared by grinding dried and de-husked areca nut using previously described protocol^3^,^27^,^56^. The powder was dissolved in 100 ml of de-ionized water at 4 °C for 4 hours with constant stirring. The solution was filtered and lyophilized. This was re-dissolved in de-ionized water and sterilized by passing through 0.2 μM filter and was re-lyophilized. Working solutions were made by dissolving the in sterile water, aliquoted and stored at −20 °C.

### Reagents and antibodies

Anti-pSMAD2/3, anti-SMAD2/3, anti-pJNK1/2, anti-JNK1/2, anti-p-c-Jun, anti-c-Jun and anti-pCAMKII primary antibodies were purchased from Cell Signalling Technology, USA. Anti-p-ATF2 and anti-ATF2 antibodies were purchased from Santa Cruz Biotechnology, USA. Anti-CAMKII antibody was purchased from Abcam and beta-actin antibody was from Sigma-Aldrich. Anti- rabbit/mouse imunnoglobulin conjugated horseradish peroxidase antibodies were from Jackson ImmunoResearch USA. Anti-mouse/rabbit immunoglobulin conjugated Alexa Fluor 488 and Alexa Fluor 647 were from Molecular Probes, ThermoFischer Scientific. TAK1 dominant negative construct was a kind gift from Prof. K.N Balaji (MCBL, Indian Institute of Science) which was obtained from Dr. Jun Nonomiya-Tsuji (North Carolina State University, Raleigh, NC).

### Cell line and treatments with pharmacological agents

HaCaT^26^,^27^ and HPL1D^57^,^58^ cell lines (immortalized keratinocytes) were used in the study and cultured as described^59^. General protocol followed for serum starvation and treatments is as follows. The cells were seeded and cultured in serum containing media for 24 hours. These cells were serum starved for the next 24 hours with three intermittent serum free washes with DMEM. Serum starved cells were then treated with the following inhibitors: Actinomycin D (100 ng/ml); Cycloheximide (10 μg/ml); TβR-I inhibitor (TGF-β type I receptor inhibitor; SB431542; 10 μg/ml); JNK inhibitor (SP600125; 10 μg/ml); MEK/ERK inhibitor (PD98059; 10 μg/ml); p38 inhibitor (SB203580; 10 μg/ml); NFkB inhibitor (Bay 11-7082; 10 μg/ml); β-catenin/Tcf inhibitor (FH535; 10 μg/ml); RGD peptide (10 μg/ml); Atropine (50 μM); KN93 (CAMKII inhibitor; 10 μg/ml); Diphenyleneiodonium (DPI; ROS inhibitor; 10 μg/ml); Rac GTPase inhibitor (EHop-016; 5 μM) for 2 hours prior to treatment with areca nut (5 μg/ml; IC_50_: 16 μg/ml^54^).

### RNA Isolation and quantitative RT- PCR

Total RNA was isolated from cells using TriZol reagent according to the manufacturer’s protocol. For RT- PCR, 2 μg of the total RNA was reverse transcribed using high capacity cDNA synthesis kit (Applied Biosystems, USA). 1/100^th^ of RT products was used per reaction for real time quantitative PCR. Gene specific primers used for the amplification of various genes by qPCR are provided in supplementary table 1.

### Enzyme Linked Immunosorbant Assay

Quantikine TGF-β2 immunoassay kit (R&D Systems) was used to detect human TGF-β2 in cell free supernatant of areca nut treated cells as per manufacter’s protocol. The O.D obtained for the conditioned media samples was interpolated on the standard curve (O.D vs Concentration) to determine concentration of TGF-β2.

### Immunoblotting

The cells were washed with chilled PBS and lysed in RIPA buffer (50 mM Tris pH 7.4; 1% NP-40; 0.25% sodium deoxycholate; 150 mM NaCl; 1 mM EDTA; 1 mM PMSF; 1 μg/ml aprotinin, leupetin and pepstatin; 1 mM sodium orthovanadate and sodium fluoride) on ice for 20 minutes. The cell debris was removed by spinning the lysate at 12000 rpm for 10 minutes at 4 °C. Total protein quantitation was done by Bradford reagent (Bio-Rad). Equal amount of protein for all samples was loaded and SDS-PAGE. The resolved proteins were transferred onto 0.4 μm PVDF membrane (Millipore). The non-specific binding on the membrane was blocked with 5% non-fat dry milk (Sigma) and probed overnight at 4 °C with primary antibody (1:1000) made in 5% TBST (20 mM Tris-HCl pH 7.4, 137 mM NaCl, 0.1% Tween-20). After washing unbound antibody the membrane was probed with anti-mouse/rabbit IgG (1:2000) for 2 hours. After washing off the non-specifically bound secondary antibody with 1X TBST; signal was captured on X-ray films (Kodak) using femtoLUCENT PLUS-HRP chemiluminescent reagent (G-Biosciences, USA).

### Immunocytochemistry

For immunocytochemistry, 50,000 cells were plated and allowed to grow onto sterile coverslips for 24 hours. Serum starved cells were then treated with various factors for two hours and fixed with 3.7% paraformaldehyde for fifteen minutes at room temperature. Paraformaldehyde was removed and washed with PBS. The cells were permeabilized by chilled methanol for 10 min at −20 °C and subsequently washed with PBS. Non-specific binding was blocked by incubating the cells with 5% BSA and 0.03% TritonX-100 in PBS for one hour at room temperature. The cells were then probed with primary antibody (1:100) overnight at 4 °C. After washing with PBS they were probed with anti-mouse/rabbit alexa fluor antibodies (1:250) for one hour at room temperature. The non-specifically bound antibody was washed away by PBS. Nuclei were stained by DAPI and the coverslips were mounted on glass slides with ProLong Gold antifade reagent (Life technologies, USA). Detection of pSMAD2 was done using Leica TCS SP5 confocal microscope (Magnification: 63X; scale bar: 5μm). Dual staining for pATF2 and p-c-Jun was detected by Zeiss LSM880Airyscan Superresolution microscopy (Magnification factor: 63X; Zoom: 1.8; Sale bar: 10 μm).

### Patient samples and Immunohistochemistry

Human tissue sections of OSF (n = 8) and normal subjects (n = 8) were obtained after informed consent from D.A Pandu R.V Dental College and Hospital after histopathological evaluation. This study was approved by the ethical committee of D.A Pandu R.V. Dental College and Hospital and was carried out in accordance with the guidelines of D.A. Pandu Dental College and hospital. To assess the activation of phospho-proteins JNK, ATF2 and Jun in OSF patients compared to normal subjects; immunohistochemistry was performed on tissue paraffin sections. The sections were deparaffinized in xylene for 15 minutes and rehydrated by passing through series of alcohol (100%, 95%, 70%; 5 minutes each) and finally immersed in double distilled water for 5 minutes. Antigen retrieval was performed in citrate buffer (pH 6) by heating the sections at in a microwave oven at 600 W for 25 minutes. The sections were cooled to room temperature, washed with 1X PBS and the intracellular peroxidase was quenched by methanol:hydrogen peroxide treatment for 20 minutes at room temperature. The sections were washed again with PBS and non-specific binding was blocked by 5% non-fat dry milk. Primary antibody (1:100) was put on the sections and incubated overnight at 4 °C. The signal was developed using the BioGenex Supersensitive polymer HRP detection kit. The sections were dehydrated and mounted using DPX. Images were captured using Zeiss microscope and processed by Zeiss Axiocam 4.3 software. Labeling index (LI) for all the stained sections was calculated using the formula (Equation 1):


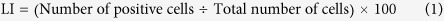


Pearson’s correlation coefficient (Pearson’s r) was calculated and plotted depicting the best fit line with 95% confidence interval using GraphPad Prism V software.

### Detection of Calcium by Fluo-4 AM

Cells were seeded in 35 mm culture dishes for 24 hours. The serum starved cells were loaded with Fluo-4, AM (ThemoFischer Scientific) in Ca^2+^ free buffer for 10 minutes at 37 °C in dark. The dye was then removed and cells were washed with PBS to remove non-internalized dye. The dish was then mounted on the microscope (Olympus IX81inverted spinning disk fluorescence microscope) and live imaging was performed with excitation at 488 nm (FITC channel). Areca nut (5 μg/ml) was added after 1 minute of capturing the basal calcium pulses and the video was monitored for a total time course of 18 minutes (Supplementary video 1).

### DCFDA assay for intracellular reactive oxygen species detection

The intracellular ROS generation upon areca nut treatment on cells was monitored by live imaging. Briefly, cells plated in 35 mm dishes were loaded with DCFDA for 10 minutes in dark and subsequently washed with PBS. The basal ROS levels were monitored for a minute and then areca nut (5 μg/ml) was added to the dish. The increase in intracellular ROS was captured in the live imaging mode of Leica TCS SP5 (live imaging mode) microscope for a total time period of 30 minutes.

### Transient transfections and knockdown

Transient transfection of shRNAs and dominant negative TAK1 was performed using Lipofectamine 2000 reagent as per manufacturer’s instructions. shRNA for JNK1, JNK2, TAK1, ATF2 and c-Jun were procured from MISSION pKLO.1 shRNA library Sigma Aldrich.

### Chromatin Immunoprecipitation

A modified protocol from Upstate Biotechnology and Sigma-Aldrich was used to perform ChIP assay. Cells were fixed with 1.42% formaldehyde for 15 minutes at room temperature. Formaldehyde was quenched using 125 mM glycine for 5 minutes at room temperature. The cells were washed and collected in 1X PBS and the cell pellet was lysed in buffer containing 50 mM Tris-HCl (pH 8.0); 200 mM NaCl; 10 mM HEPES (pH 6.5); 0.1% SDS; 10 mM EDTA; 0.5 mM EGTA; 1 mM PMSF; 1 μg/ml of leupeptin, pepstatin and aprotinin; 1 mM sodium orthovanadate and NaF. Bioruptor Plus (Diagenode) was used to shear chromatin at high power for 65 rounds (30 second pulse on/45seconds pulse off) at 4 °C to obtain DNA fragments of average size 200 to 500 bp. This was immunoprecipitated using 1 μg/ml antibody for pATF2 or p-c-Jun or anti-rabbit IgG and the immune complexes were pulled down using rprotein A-sepharose Fast Flow beads (GE Healthcare Biosciences). These were washed with wash buffer A (50 mM Tris-HCl (pH 8); 500 mM NaCl; 1 mM EDTA; 1% Triton X-100; 0.1% sodium deoxycholate; preatease and phosphatase inhibitors), B (50 mM Tris HCl (pH 8); 1 mM EDTA; 250 mM LiCl; 0.5% NP-40; 0.5% sodium deoxycholate, phosphatase and protease inhibitors), and TE (10 mM Tris HCl (pH 8); 1 mM EDTA with protease and phosphatase inhibitors) sequentially. The precipitated DNA was then eluted in elution buffer (1% SDS; 0.1M sodium bicarbonate) at 65 °C for 30 minutes and subsequently de-crosslinked using 200 mM NaCl. The sample was then treated with RNase and Proteinase K followed by phenol-chloroform precipitation. The purified DNA was analyzed for pATF2 and p-c-Jun binding sites on TGF-β2 promoter (NCBI Reference Sequence: NG_027721.1)^39^ by performing real time PCR with site specific primers, (F) 5′ATTTCCACACTCCCTCAACG 3′ (R) 5′TCTCTGAACCACGTGTCTGC3′. The result obtained was normalized to the input and IgG pulldown values and was plotted as fold enrichment.

### Statistical Analysis

The graphs represent mean ± standard error of mean and comparison between multiple data sets was done by one way analysis of variance (ANOVA). Unpaired t-test was performed to assess statistical significance for the median labeling index for staining of various protein molecules in normal and OSF tissue samples. GraphPad Prism V software was used to analyze data for statistical significance. p value ≤ 0.01, 0.001, 0.0001 are represented as *^,^**^,^*** respectively.

## Additional Information

**How to cite this article**: Pant, I. *et al*. Role of areca nut induced JNK/ATF2/Jun axis in the activation of TGF-β pathway in precancerous Oral Submucous fibrosis. *Sci. Rep.* **6**, 34314; doi: 10.1038/srep34314 (2016).

## Supplementary Material

Supplementary Figures

Supplementary Information
