# Supplementary material for: Role of areca nut induced JNK/ATF2/Jun axis in the activation of TGF-β pathway in precancerous Oral Submucous Fibrosis
Source: Sci Rep. 2016 Oct 6;6:34314. doi: 10.1038/srep34314 (PMC5052620; doi:10.1038/srep34314)
Supplement: Supplementary Information [file srep34314-s2.pdf]

Role of areca nut induced JNK/ATF2/Jun axis in the activation of TGF-β pathway in precancerous Oral Submucous Fibrosis

Ila Pant[1], S. Girish Rao[2], Paturu Kondaiah[1]*

1. Department of Molecular Reproduction, Development and Genetics, Indian Institute of Science, Bangalore- 560012, India

2. Department of Oral and Maxillofacial Surgery D.A Pandu Memorial- R.V Dental College and Hospital, Bangalore-560078, India

*Corresponding Author:

Paturu Kondaiah

E-mail: paturu@mrdg.iisc.ernet.in; Tel.: +91-80- 22932688, Fax: +90-80-23600999

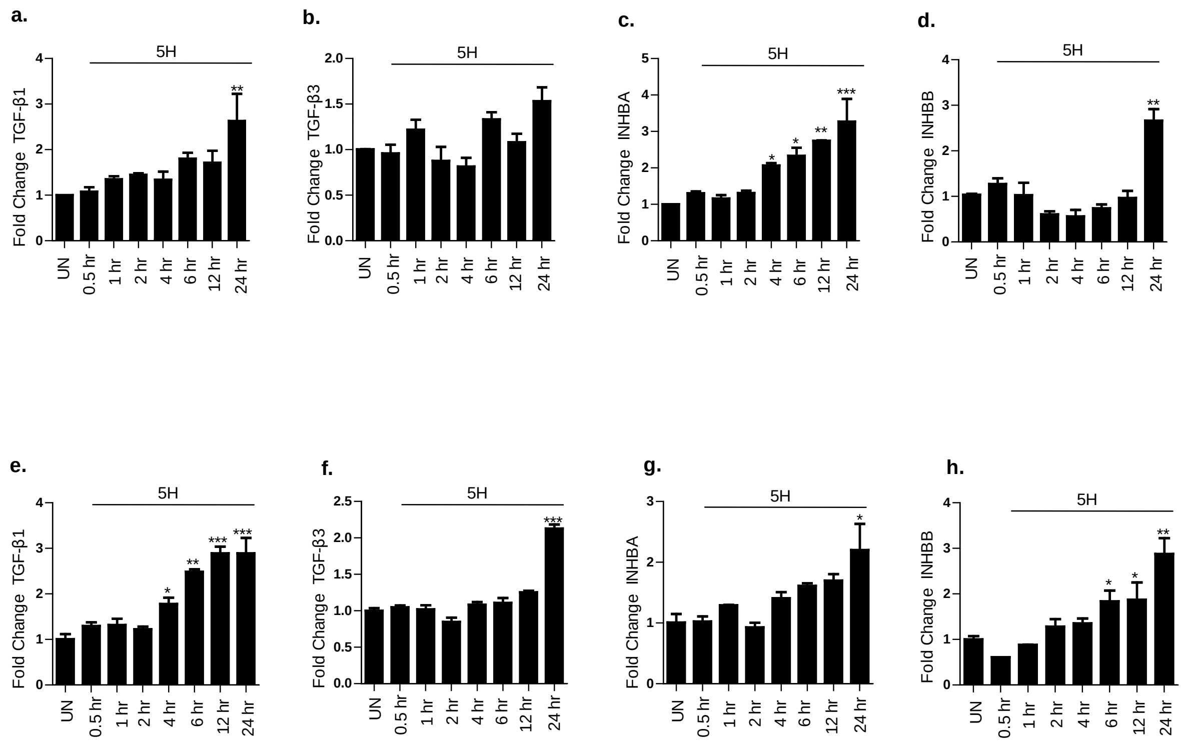

Supplementary figure 1. Regulation of TGF-β superfamily members by areca nut in epithelial cells.

qRT-PCR data representing fold change in TGF-β1 (a, e), TGF-β3 (b, f), INHBA (c, g) and INHBB (d, h) transcripts in HaCaT (a-d) and HPL1D (e-h) cells upon areca nut treatment (5H; 5 μg/ml) at various time points compared to untreated cells. ***, **,* represent p values ≤ 0.0001; 0.001; 0.01 respectively.

a.

b.

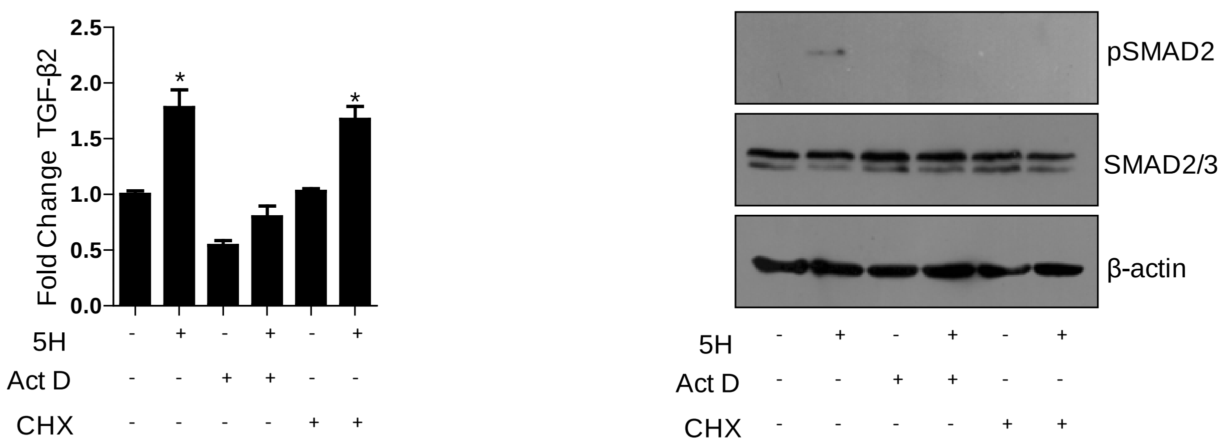

Supplementary figure 2. Areca nut mediated activation of TGF-β pathway in HPL1D cells. a & b) TGF-β2 transcript (bar graph) and pSMAD2 (immunoblot) levels upon 2 hour treatment with areca nut (5H; 5 µg/ml) with and without actinomycin D (Act D) or cycloheximide (CHX) treatment. β-actin is used as loading control for immunoblots. * represents p value ≤ 0.01.

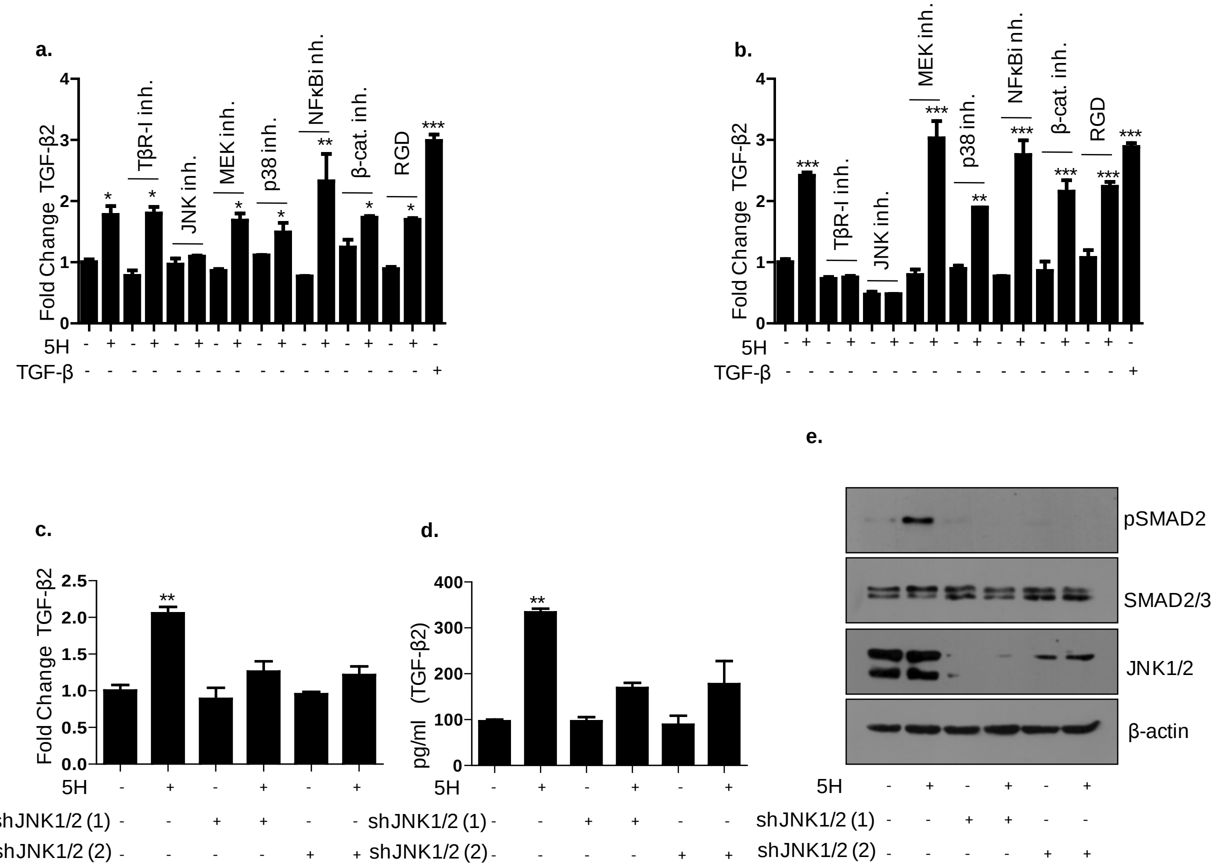

Supplementary figure 3. Areca nut induced TGF-β pathway activation is dependent on JNK in HPL1D cells.

a & b, are bar graphs representing fold change of TGF-β2 transcript at 2 and 24 hours respectively by 5H treatment with or without the indicated inhibitors. TGF-β treatment is used as positive control. Bar graphs depicting TGF-β2 transcript fold change (c) and protein (d) at 2 hours upon transient knockdown of JNK1/2 by using two different combinations of shRNAs (1 & 2) with or without areca nut treatment. e) Immunoblot representing pSMAD2 at 2 hours upon transient knockdown of JNK1/2 using two different combinations of shRNAs (1 & 2) with or without areca nut treatment. β-actin is used as loading control for immunoblots. ***, **,* represent p values ≤ 0.0001; 0.001; 0.01 respectively. (5H; 5 µg/ml areca nut extract).

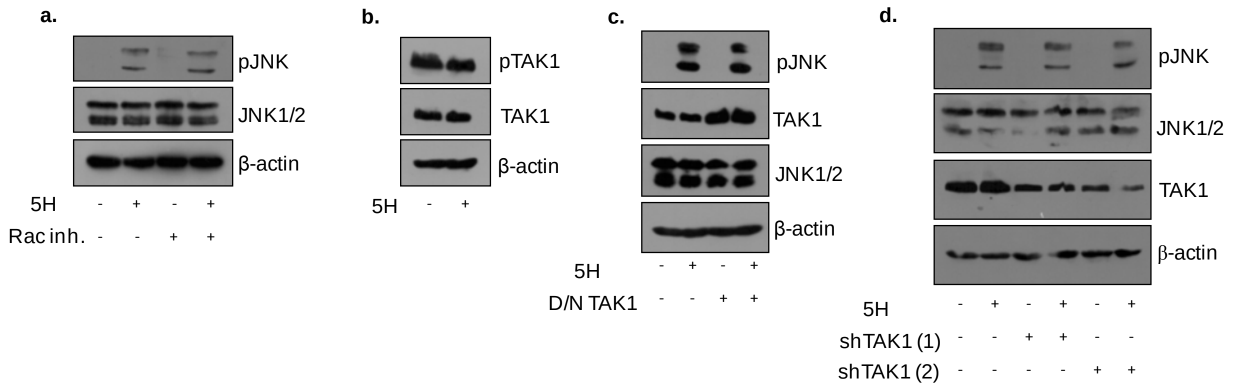

Supplementary figure 4. Areca nut induced JNK activation is independent of Rac and TAK1 activity in HaCaT cells.

a) Immunoblot representing pJNK levels which are not compromised in the presence of Rac-GTPase inhibitor at 30 minutes. b) Immunoblot showing that areca nut does not regulate pTAK1 levels at 30 minutes. c & d) Immunoblot representing pJNK levels which are not compromised by over-expression of dominant negative TAK1 (D/N) or upon transient knock down of TAK1. β-actin is used as loading control for immunoblots. (5H; 5 μg/ml areca nut extract).

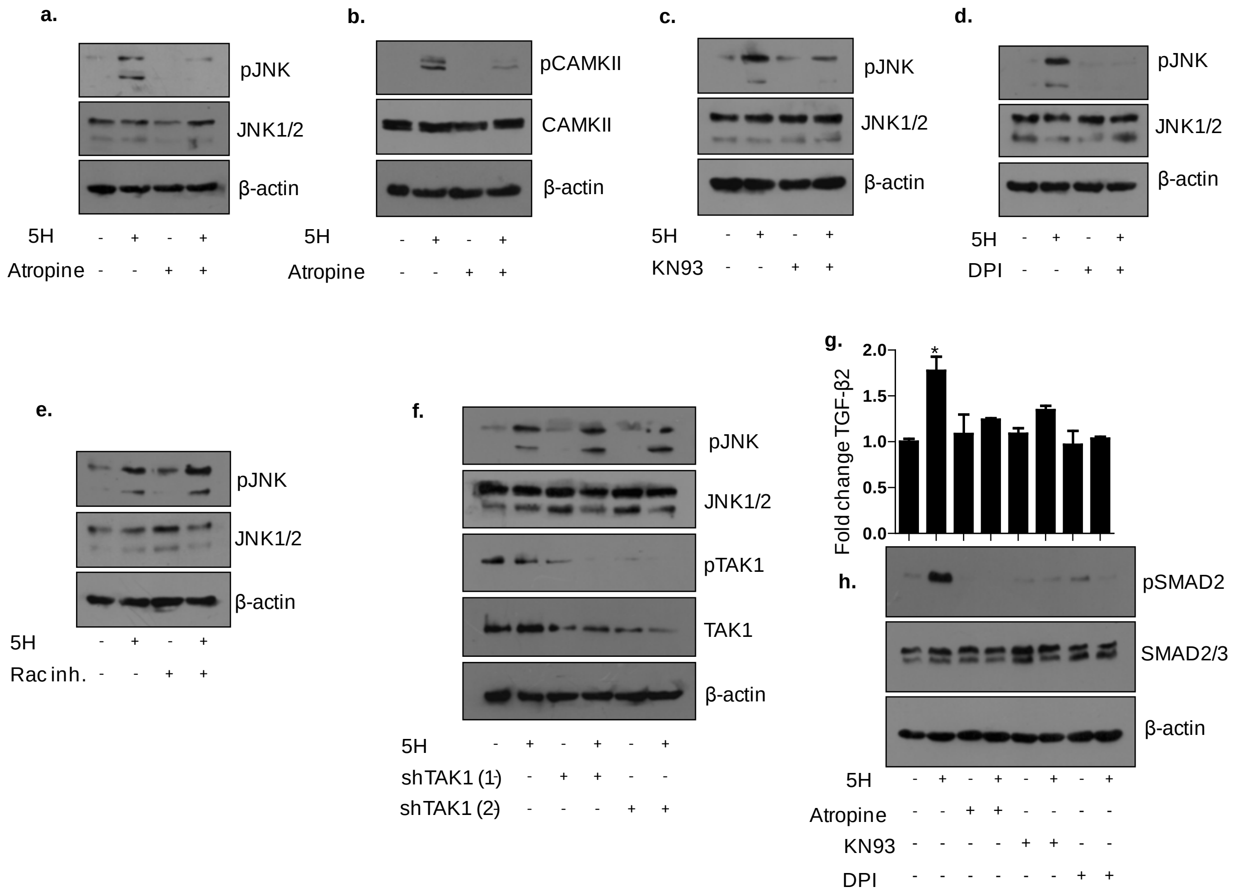

Supplementary figure 5. Activation of JNK/TGF-β axis by areca nut is dependent on Ca^{2+}/CAMKII and ROS in HPL1D cells.

a) Immunoblots representing compromise in areca nut induced pJNK levels upon treatment with atropine at 30 minutes. b) Immunoblots representing compromise in areca nut induced pCAMKII levels upon treatment with atropine at 30 minutes. c & d) Immunoblots representing compromise in areca nut induced pJNK levels upon treatment with KN93 and DPI at 30 minutes respectively. e) Immunoblots representing areca nut induced pJNK levels are not compromised by Rac-GTPase inhibitor at 30 minutes. f & g) Immunoblots representing areca nut induced pJNK levels at 30 minutes which are not compromised by transient knock down of TAK1. g & h) Bar graph and immunoblots showing regulation of TGF-β2 transcript and pSMAD2 respectively, at 2 hours by areca nut, which are compromised upon treatment with atropine,

KN93 and DPI. β-actin is used as loading control for immunoblots. * represents p value ≤ 0.01. (5H; 5 µg/ml areca nut extract).

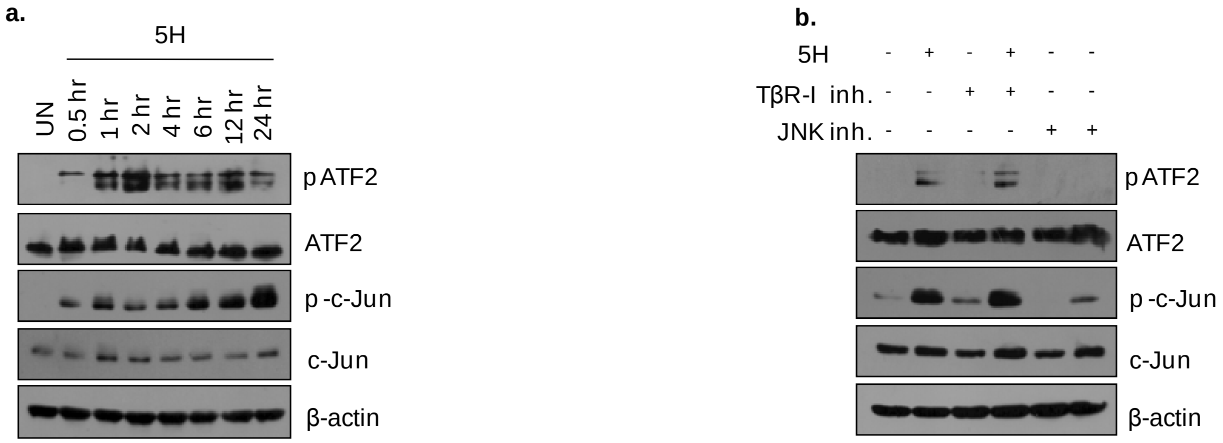

Supplementary figure 6. Areca nut activates ATF2 and c-Jun in HPL1D cells.

a) Immunoblots representing areca nut induced phosphorylation of ATF2 and c-Jun at the indicated time points. b) Immunoblots representing areca nut induced pATF2 and p-c-Jun at 30 minutes which are sustained even in the presence of TβR-I inhibitor but get compromised in the presence of JNK inhibitor. β-actin is used as loading control for immunoblots. (5H; 5 µg/ml areca nut extract).

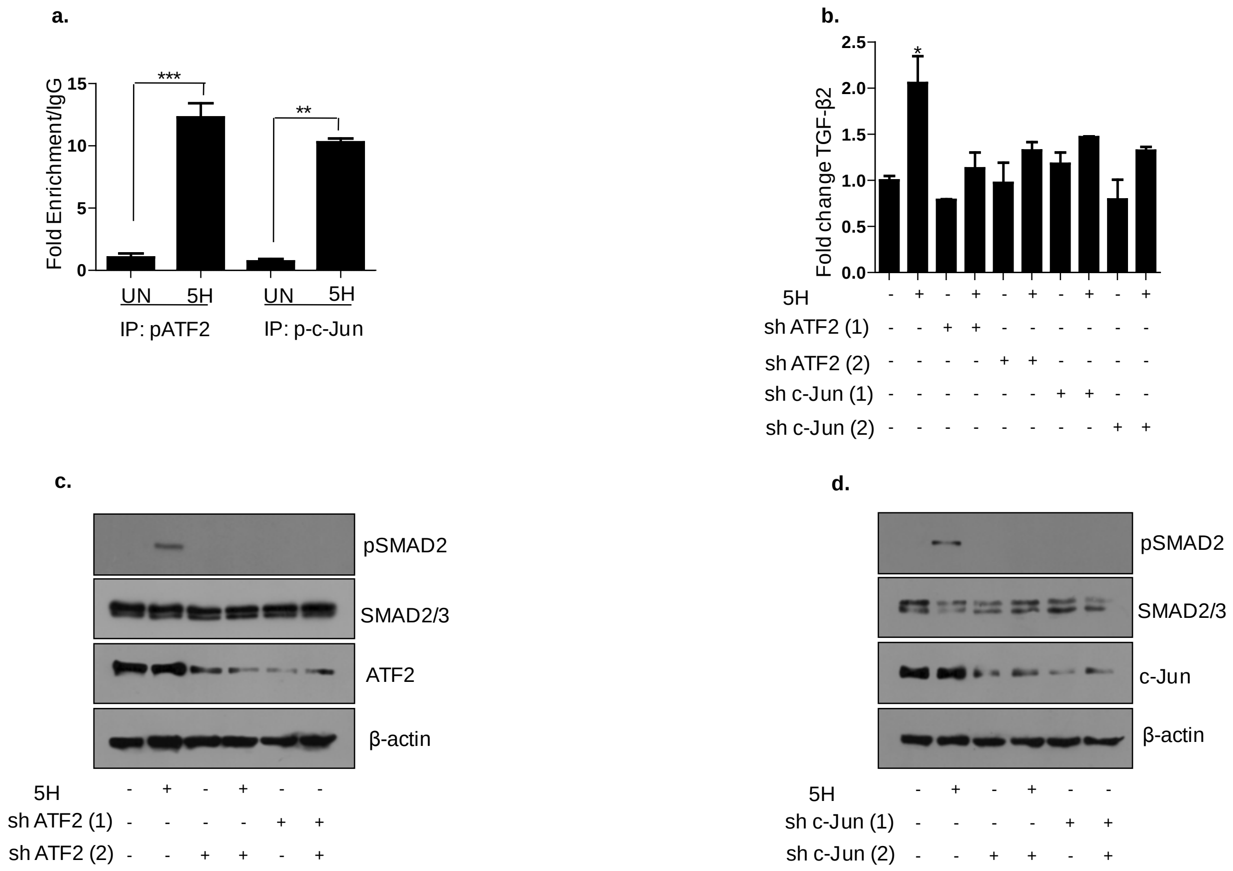

Supplementary figure 7. Areca nut induced TGF-β pathway activation is mediated by ATF2 and c-Jun in HPL1D cells.

a) qPCR graph of chIP assay showing fold enrichment of pATF2 and p-c-Jun on TGF-β2 promoter at 2 hours upon areca nut treatment compared to untreated cells. b) Bar graph showing fold change at 2 hour of areca nut induced TGF-β2 and its compromise upon transient knock down of ATF2 or c-Jun. c & d) Immunoblots representing pSMAD2 levels at 2 hours of areca nut treatment and its compromise upon transient knock down of ATF2 or c-Jun. β-actin is used as loading control for immunoblots. ***, **, * represent p values ≤ 0.0001; 0.001; 0.01 respectively. (5H; 5 µg/ml areca nut extract).

Supplementary Video 1. Areca nut induces calcium mobilization.

Video of Fluo 4-AM calcium detection assay showing increase in calcium levels upon areca nut treatment over a period of 18 minutes. Treatment with areca nut extract (5 µg/ml) was given post 1 minute of capturing basal calcium levels in the cells. (Video made at the rate of 10 seconds per frame captured)

Supplementary Table 1. List of gene specific primers used for qPCR

No.	GENE	FORWARD PRIMER 5'-3' sequence	REVERSE PRIMER 5'-3' sequence	DETAILS
1	INHBA	AATCTCGAAGTGCAGCGTCT	GGAGAACGGGTATGTGGAGA	133 bp, 60°C
2	INHBB	GCGTTTCCGAAATCATCAG	TTTCAGGTAAAGCCACAGGC	134 bp, 59.5°C
3	RPL35A	GAACCAAAGGGAGCACACAG	CAATGGCCTTAGCAGGAAGA	236 bp, 58°C
4	TGFβ1	TCCGAGAAGCGGTACCTGAA	TGCTGTCACAGGAGCAGTGG	266 bp,63.7°C
5	TGFβ2	AGTGCCTGAACAACGGAT	GTACAAAAGTGCAGCAGG	218 bp, 55°C
6	TGFβ3	GCGTGAGTGGCTGTTGAGA	CCAAGTTGCGGAAGCAGTA	306 bp,52.7°C
